# Tobacco Rattle Virus as a Tool for Rapid Reverse-Genetics Screens and Analysis of Gene Function in *Cannabis sativa* L.

**DOI:** 10.3390/plants11030327

**Published:** 2022-01-26

**Authors:** Hanan Alter, Reut Peer, Aviv Dombrovsky, Moshe Flaishman, Ben Spitzer-Rimon

**Affiliations:** 1 Institute of Plant Sciences, Agricultural Research Organization—Volcani Institute, HaMaccabbim Road 68, Rishon LeZion 7505101, Israel; hanana@volcani.agri.gov.il (H.A.); reutp@volcani.agri.gov.il (R.P.); vhmoshea@volcani.agri.gov.il (M.F.); 2 Department of Plant Science, The Robert H. Smith Faculty of Agriculture, Food and Environment, The Hebrew University of Jerusalem, P.O. Box 12, Rehovot 7610001, Israel; 3 Department of Plant Pathology and Weed Research, Agricultural Research Organization—Volcani Institute, HaMaccabbim Road 68, Rishon LeZion 7505101, Israel; aviv@volcani.agri.gov.il

**Keywords:** cannabis, PDS, TRV, VAGE, VIGS

## Abstract

Medical cannabis (*Cannabis sativa* L.) is quickly becoming a central agricultural crop as its production has continued to increase globally. The recent release of the cannabis reference genomes provides key genetic information for the functional analysis of cannabis genes. Currently, however, the established tools for in vivo gene functional analysis in cannabis are very limited. In this study, we investigated the use of the tobacco rattle virus (TRV) as a possible tool for virus-induced gene silencing (VIGS) and virus-aided gene expression (VAGE). Using leaf photobleaching as a visual marker of *PHYTOENE DESATURASE* (*PDS*) silencing, we found that VIGS was largely restricted to the agro-infiltrated leaves. However, when agro-infiltration was performed under vacuum, VIGS increased dramatically, which resulted in intense *PDS* silencing and an increased photobleaching phenotype. The suitability of TRV as a vector for virus-aided gene expression (VAGE) was demonstrated by an analysis of DsRed fluorescence protein. Interestingly, a DsRed signal was also observed in glandular trichomes in TRV_2_-*DsRed*-infected plants, which suggests the possibility of trichome-related gene function analysis. These results indicate that TRV, despite its limited spread, is an attractive vector for rapid reverse-genetics screens and for the analysis of gene function in cannabis.

## 1. Introduction

*Cannabis sativa* L. (cannabis) is an important medical and industrial crop; its use continues to expand as its consumption for both medicinal and recreational use increases [1]. Known as one of the oldest cultivated plants, cannabis originated in Central Asia and then spread throughout Asia and Europe [2,3,4]. The genus *Cannabis* is part of the Cannabaceae family, and despite controversy regarding the number of species comprising the genus, the most accepted assumption is that *Cannabis* is a monotypic genus (*C. sativa* L.) with three sub-species: sativa, indica, and ruderalis [2,3,4,5]. Regardless of the differences in phenotypic appearance and chemical profile among the subspecies, intensive crossbreeding has resulted in the fading of the classical unique characteristics of each population [1,6].

Cannabis plants are mostly dioecious and produce either male or female flowers. Although sex determination appears to be controlled by a system of sex chromosomes, non-optimal growing conditions and changes in environmental factors, such as photoperiod and temperatures, are known to cause the development of male or hermaphroditic flowers [7,8,9,10]. The medical cannabis distributed commercially uses only vegetatively propagated female cannabis plants [5,6] because most of the specialized metabolites responsible for the medicinal properties of the cannabis plant are present in high levels, within the trichromes concentrated on mature non-fertilized female inflorescences [10,11,12]. These compounds include numerous phytomolecules such as cannabinoids, terpenoids, and flavonoids that are known for their unique medicinal properties [6]. In recent years, the consumption of cannabis for medical purposes has increased following new discoveries regarding the effectiveness of cannabis treatment for a vast variety of medical conditions [13,14]. Therefore, it is of great importance to identify and characterize the genetic factors regulating the attributes that affect the value of the cannabis crop, such as quality and quantity of cannabinoids, sex determination, resistance to biotic and abiotic stresses, and the mechanism responsible for flowering. 

Recently, expanded genomic and transcriptomic resources have been used to identify the candidate genes participating in and regulating value-related traits [7,9,15,16,17,18,19,20,21]. These genomic data have provided new insights into the chromosome arrangement of cannabinoid biosynthetic genes that support a model wherein *TETRAHYDROCANNABINOLIC ACID SYNTHASE* and *CANNABIDIOLIC ACID SYNTHASE* are two different genes located in close proximity, not two co-dominant alleles at a single locus [9,19,20,22]. The transcriptomic analysis of segregating populations and the mapping of sex related transcripts to the available cannabis genome were used to identify the sex chromosomes in cannabis [8,9]. Despite this considerable progress in understanding cannabis genetics and the increase in available genomic resources, the lack of well-established tools for in vivo gene functional analysis is one of the major obstacles hindering cannabis research at the molecular level. One method used with some success is the combination of ethyl methanesulfonate (EMS) with the TILLING screening-technique to identify mutations in *FATTY ACID DESATURASE* genes in hemp, which resulted in the modification of the seed-oil composition [23]. The requirement for large populations and the generation of homozygous mutations in dioecious plants limits the harnessing of EMS mutagenesis and TILLING methods for functional genomics in cannabis, particularly because of security regulatory issues and logistical challenges. 

Cannabis is considered a recalcitrant plant, and stable transformation is considerably challenging. Recently, *Agrobacterium*-mediated gene transformation was used to generate transgenic hemp lines expressing *Β-GLUCURONIDASE* using different seedling organs as explants [24]. In addition, the generation of stable transgenic plants demonstrated successful CRISPR/Cas9-based technology gene editing of *PHYTOENE DESATURASE* (*PDS*) in cannabis [25]. Despite these breakthroughs, it seems that additional effort is required to increase the efficiency of transformation and gene editing, which is relatively low. The use of seedlings as explants may create challenges for further genetic analysis since cannabis varieties have considerably high heterozygosity levels [21]. Nonetheless, functional characterization of cannabis genes will accelerate once stable transformation approaches become well established [7]. As a very efficient alternative for stable transformation in other crops, virus-induced gene silencing (VIGS) and virus-aided gene expression (VAGE) have been used for gene functional analyses and for extensive reverse genetics screens in a variety of plants, including petunias, tomatoes, *Arabidopsis*, and strawberries [26,27,28,29,30,31,32]. These well-established approaches involve the viral inoculation of target plants using a viral vector, cloned in a binary vector, to induce gene silencing or, alternatively, gene expression. Among several viral vectors available, one of the most commonly used is the tobacco rattle virus (TRV)-based vector. This efficient vector is characterized by the broad spectrum of hosts it can infect, its relatively high silencing efficiency, and the mild symptoms caused by its infection [26,27,28,29,30,31]. TRV has been used to investigate the functionality of several genes involved in the regulation of specialized metabolites, disease resistance, plant development, and abiotic stress in many plant species, including recalcitrant plants [26,27,28,29,30,31]. In cannabis, *cotton leaf crumple virus* (CLCrV) was recently used for VIGS by inoculation of cotyledons and young seedlings. Silencing *PDS* and *MAGNESIUM CHELATASE SUBUNIT I* (*ChlI*) using CLCrV silenced ~70% of the target genes [33]. However, the strong leaf photobleaching phenotype usually attained following *PDS* and *ChlI* silencing using a TRV vector was not observed in cannabis; only faint green leaves with white and yellow spots were observed [33]. 

In this study, we investigated the use of *PDS* silencing and *DsRed* overexpression along with TRV-based VIGS and VAGE as tools for the functional analysis of genomics in cannabis. This is the first report of virus vector-based gene expression and gene silencing that generated a clear distinguishable phenotype in cannabis. Our results suggest that TRV would be useful as a platform for rapid reverse-genetics screens and for analysis of gene function. In light of the relatively limited genetic tools available, the use of TRV-based VIGS is an attractive approach for the functional analysis of genomics in cannabis.

## 2. Results and Discussion

### 2.1. VIGS in Cannabis Using a TRV Vector 

**Figure 1:**
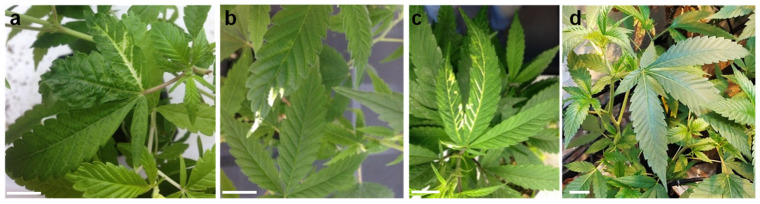
TRV_2_-based VIGS of *PDS* in different medical cannabis lines. Cannabis plantlets from line MF-71 (**a**), line MF-169 (**b**), and line MF-219 (**c**) 2 weeks after agro-infiltration using a syringe. A mixture of *Agrobacterium* transformed with pTRV_1_ and pTRV_2_ (**d**) or pTRV_2_ carrying the 310 bp fragment of *PDS* (**a**–**c**) was used (scale bar = 2 cm).

To evaluate TRV as a VIGS vector in cannabis, we targeted its *PDS* gene by cloning a 310 bp fragment in the center of the mRNA (base pairs 913 to 1222 of the total 2202 bp) into pTRV_2_ (pTRV_2_-*PDS*_310_). *PDS* silencing is widely used as a marker for gene silencing due to the easily recognizable photobleaching phenotype it produces in leaves [26,27,28,29,30,31]. Since different responses to TRV infection among cultivars of the same species have been reported [27,29,34], we selected cuttings from three genetic lines (MF-71, MF-169, and MF-219) from the breeding program at the ARO, Volcani Institute for the initial evaluation of TRV-based VIGS in cannabis. Using a needleless syringe, a culture mixture of *Agrobacterium tumefaciens* carrying pTRV_1_- and pTRV_2_-*PDS*_310_ vectors was infiltrated to the abaxial side of the leaves to produce the TRV infection. Two weeks after inoculation, photobleaching was observed in all cannabis lines tested (Figure 1a–c) but not in the control plants infected with the pTRV_2_ vector (Figure 1d). The photobleaching noted in all lines was local and observed mainly in the leaf veins. Among the different lines, MF-219 demonstrated the strongest and most widely spread photobleaching in four out of five inoculated plants. The photobleaching in MF-169 and MF-71 was relatively mild and appeared only in two and three out of five inoculated plants, respectively (Figure 1). In light of these results, the MF-219 line was selected for further studies. 

**Figure 2:**
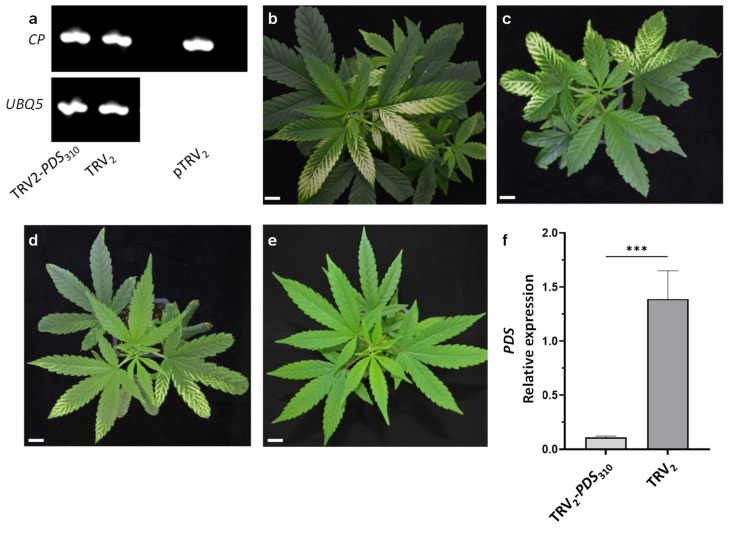
A comparison of different TRV_2_-*PDS* vectors inoculated using vacuum infiltration. (**a**) The detection of the viral coat protein (CP) transcripts by RT-PCR in the cannabis line MF-219 infiltrated with pTRV_2_-PDS_310_ or pTRV2, and on pTRV_2_ plasmid as a positive control. UBQ5 was used as a control gene. Cannabis plantlets inoculated with pTRV_1_- and pTRV_2_-*PDS*_310_ (**b**), pTRV2-*PDS*_486_ (**c**), pTRV_2_-*PDS*_424_ (**d**), or pTRV_2_ (**e**) 15 days after vacuum infiltration (scale bar = 2 cm). (**f**) A quantitative real-time PCR analysis of *PDS* transcript levels in cannabis leaves infected with TRV_2_ and TRV_2_-*PDS*_310_. The data were normalized to *UBQ5* with the standard error indicated by vertical lines. The significant difference between treatments (*p* ≤ 0.0006; *n* ≥ 3; ***) was calculated using Student’s *t* test.

VIGS efficiency depends on the gene sequence used and the number of TRV-infected cells. In an effort to increase the efficiency of agro-infection in cannabis, a vacuum-based infiltration protocol was used. Vacuum infiltration has been shown to be more efficient when compared to leaf infiltration using a syringe, especially in plants with less permeable leaves [35]. Therefore, 3-week-old rooted cuttings were infiltrated with *Agrobacterium* culture using a vacuum chamber. In addition, two other *PDS* fragments that included 424 bp from the 5′ end (TRV_2_-*PDS*_424_) and 486 bp from the 3′ end (TRV_2_-*PDS*_486_) were cloned into pTRV_2_. This allowed us to assess whether the position of the DNA fragment selected for inducing the *PDS* silencing would affect the silencing efficiency, as previously reported [36]. The first signs of photobleaching were noticed at 7 days post-vacuum infiltration. To validate the infection of TRV_2_ in tissue inoculated with pTRV_2_-*PDS*_310_, RT-PCR was performed using TRV_2_ *COAT PROTEIN* (*CP*)-specific primers. The presence of the *CP* amplicon indicated TRV_2_ infection (Figure 2a). 

After an additional week, the photobleaching phenotype was clearly widespread in most of the plants (four out of five plants for each construct) of the vacuum-infiltrated compared to the syringe-infiltrated leaves (Figure 1 and Figure 2b–d). The photobleached leaf area was clearly greater in cannabis plants that were inoculated with the pTRV_2_-*PDS*_310_ and pTRV_2_-*PDS*_486_ vectors (Figure 2b,c) than in plants inoculated with the pTRV_2_-*PDS*_424_ vector (Figure 2d). These results are similar to data from previous reports that demonstrated variation among different constructs used to induce VIGS of a specific gene [36]. To estimate *PDS* silencing in the photobleached tissues, its transcript levels relative to those of *UBIQUITIN 5* were determined using quantitative real-time PCR. The *PDS* transcript level in photobleached leaves of plants infected with TRV_2_-*PDS*_310_ was lower by more than 90% compared to the TRV_2_ infected leaves from control plants (Figure 2e,f). These results indicated that the *PDS* transcript level was downregulated in the photobleached tissue as a result of TRV_2_-*PDS*_310_ infection. 

Systemic silencing using TRV seems to be relatively limited in cannabis. Local photo-bleaching in plants inoculated using syringe infiltration or in newly emerging leaves using vacuum infiltration indicated that the infection was localized, suggesting that both TRV cell-to-cell and systemic movement were limited, particularly the latter. In contrast, the use of VIGS with the CLCrV vector in cannabis seems more efficacious systemically, since the silencing was observed on newly emerging leaves that were not infected [33]. However, *PDS* silencing using CLCrV yielded only faint green leaves with white and yellow spots in contrast to the complete photobleaching observed using TRV in our study [33]. 

The uneven local spread of virus vectors throughout the infected plant is a major limitation of the VIGS system that hinders gene functional analysis in plants. However, co-silencing of the gene of interest and marker genes overcomes such limitations. A number of marker genes, such as *CHALCONE SYNTHASE* and *ANTHOCYANIN 2*, as well as transgene silencing of *GREEN FLUORESCENT PROTEIN* and *DELILA* and *ROSEA1*, which result in visible phenotypes upon silencing, have been used to identify the affected tissues. These approaches led to identification and characterization of genes involved in senescence, specialized metabolism, and fruit ripening, for example [27,34,37,38,39,40]. Therefore, the clearly distinguishable phenotype and significant *PDS* silencing that occurred only 2 weeks after infection indicate the feasibility of using TRV-based VIGS for easy, efficient, and fast gene functional analysis in cannabis.

### 2.2. VAGE in Cannabis Using a TRV Vector

**Figure 3:**
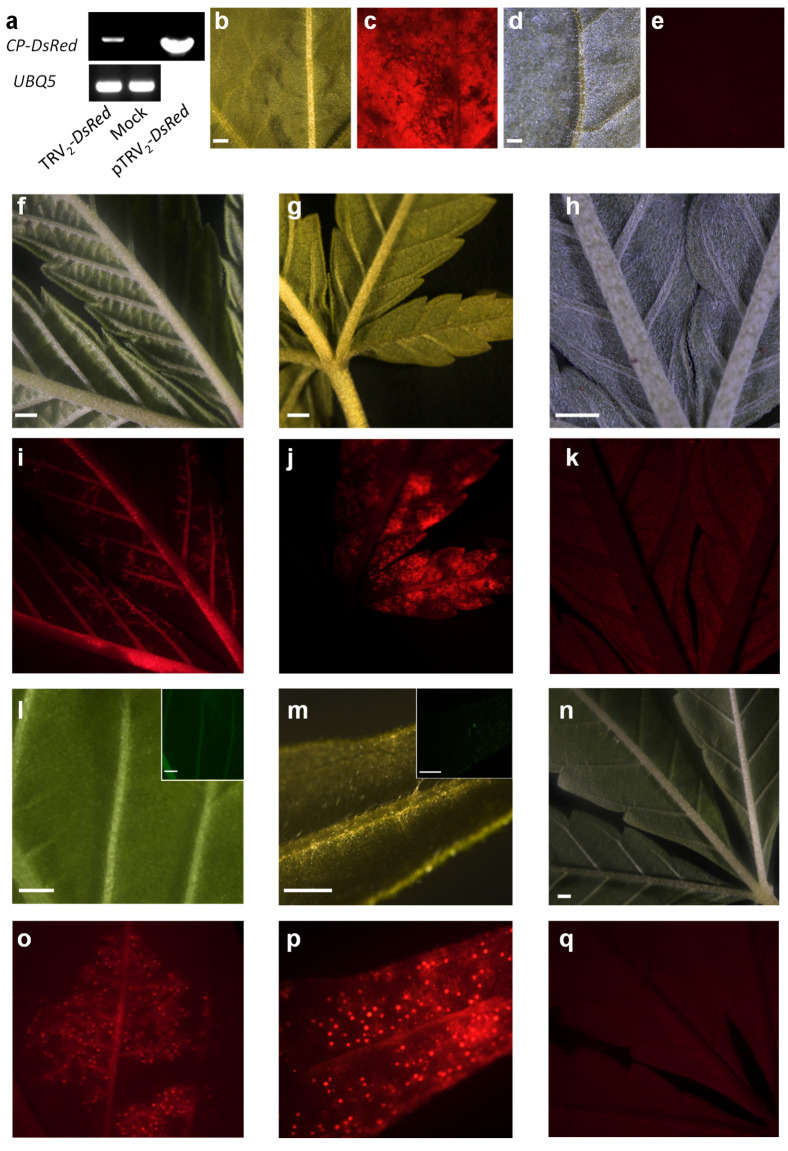
TRV_2_-mediated expression of *DsRed*. (**a**) Detection of the viral CP-*DsRed* transcripts by RT-PCR in cannabis line MF-219 2 weeks after inoculation with pTRV_2_-*DsRed* or mock inoculated plants and on pTRV2-*DsRes* plasmid as a positive control. UBQ5 was used as a control gene. *Nicotiana benthamiana* (**b**,**c**) and cannabis (**f**,**i**,**l**,**o**) inoculated with pTRV_2_-*DsRed* by syringe infiltration or by vacuum infiltration (**g**,**j**,**m**,**p**). pTRV_2_-inoculated *Nicotiana benthamiana* (**d**,**e**) and cannabis (**h**,**k**) were used as controls in addition to mock-inoculated cannabis (**n**,**q**). The images were taken using a fluorescence stereomicroscope using red (**c**,**e**,**i**–**k**,**o**–**q**) and bright field (**b**,**d**,**f**–**h**,**l**–**n**) channels. The insets in (**i**,**m**) are the same images taken in the GFP channel (scale bar = 1 mm).

To evaluate the viability of TRV as a vector for VAGE in cannabis, we used pTRV_2_ containing the ORF of the *DsRed* fluorescent protein (pTRV_2_-*DsRed*) [28,29,41]. Vacuum and syringe agro-infiltration were used as inoculation methods, similar to the approach used for the inoculation of pTRV_2_-*PDS* vectors. Two weeks after inoculation, TRV_2_ infection was confirmed using RT-PCR performed with forward and reverse primers complementary to pTRV_2_ *CP* and *DsRed*, respectively (Figure 3a). The presence of the *CP-DsRed* amplicon indicated that TRV_2_ infection occurred. No amplification was observed in the mock inoculated plants used as a negative control (Figure 3a). The DsRed signal, which was analyzed under a fluorescence stereomicroscope, was detected in *Nicotiana benthamiana*, which was used as positive control, as well as in the cannabis plants inoculated using both syringe and vacuum agro-infiltration (Figure 3). Similar to infection with TRV_2_-*PDS*, it seems that the systemic spread of TRV was relatively limited. However, the DsRed signal could also be detected in leaves that were not directly inoculated by the agro-infiltration. Interestingly, the DsRed signal was also observed in the glandular trichomes of TRV_2_-*DsRed*-infected plants (Figure 3o,p). The fluorescence signal in the glandular trichomes appears to have originated in DsRed proteins and not from auto-fluorescence signal, as it was specific to the red channel and not detected in the GFP channel (Figure 3i,m). Moreover, it was not uniformly distributed in the leaf but rather localized only in regions showing the DsRed signal in the surrounding tissues. No signal was observed in control plants inoculated with pTRV_2_ or mock inoculated (Figure 3d,e,h,k,n,q). These results suggest that it is most likely that TRV accommodates the glandular trichomes, which demonstrates the possibility to analyze trichomes related gene functions. Collectively, the strong *DsRed* expression observed in TRV-*DsRed*-infected plants provides compelling evidence that TRV can be used as an efficient vector for expressing genes of interest. In addition, these results extend the set of genetic tools available for functional genomics analysis in cannabis.

In recent years, much time and effort has been invested in developing transgene-free genome-modified plants using viral vectors [41,42,43,44,45]. Site-directed mutagenesis of heritable mutations in petunia and ornamental tobacco plants were generated using TRV-expressing genome editing components such as meganuclease and zinc finger nucleases [41,44]. TRV has also been used successfully for sgRNA delivery in Cas9-overexpressing transgenic plants [42,43]. The size constraints of the TRV viral vector limit the delivery of large genes such as CRISPR-Cas9. However, it is likely that the continuous development of compact Cas proteins will make it possible in the near future to use TRV to deliver all CRISPR reagents required for genome editing similar to the way adeno-associated viruses are used in human cell lines [46,47,48]. 

We propose TRV as a platform for rapid reverse-genetics screens and for analysis of gene function. The field of cannabis research will benefit greatly from the implementation and expansion of genetic tools for gene functional analysis. TRV has the advantage of requiring only a short time to induce silencing or to express a gene of interest. Given the limited genetic tools available, the use of TRV-based VIGS is an attractive approach with which to explore gene function in cannabis.

## 3. Materials and Methods

### 3.1. Plant Material

Three high-THC, low-CBD medical *Cannabis* cultivars (MF-219, MF-169, and MF-71) that originated in the ARO, Volcani Institute cannabis breeding program were vegetatively propagated using cuttings. Rooted cuttings were grown in 200 mL pots for 3 weeks under a long-day photoperiod (18/6 h light/dark) in an environmentally controlled growth chamber at a constant temperature of 22 °C before and after infection with TRV.

### 3.2. Construction of pTRV_2_ Vectors 

To generate pTRV_2_-*PDS*_310_, which contains 310 bp corresponding to base pairs 967 to 1296 of PDS mRNA (XM_030651587.1), a DNA fragment with the addition of Xhol and XbaI recognition sites at the 5′ and 3′ ends, respectively, was synthesized and cloned into pUC57 to generate pUC57-*PDS*_310_ (IDT, Coralville, IA, USA). *PDS*_424_ and *PDS*_486_ fragments were generated using PCR reactions using Kodaq 2X PCR MasterMix (ABM, Richmond, Canada) and primer sets 1 and 2 (Table 1), carrying Xhol and XbaI restriction enzyme recognition in the forward and reverse primers, respectively. pUC57-*PDS*_310_, *PDS*_486_, and *PDS*_424_ fragments were digested by Xhol and XbaI (NEB, Ipswich, MA, USA); further ligated into pTRV_2_ [27,28,29]; and digested with the same enzymes to generate pTRV_2_-*PDS*_310_, pTRV2-*PDS*_486_, and pTRV2-*PDS*_424_, respectively. Finally, all constructs were transformed into *Agrobacterium* (GV3101 strain). 

### 3.3. Agro-Inoculation of pTRV Vectors

For the inoculation of *Agrobacterium,* separate 10 mL (for syringe infiltration) or 500 mL (for vacuum infiltration) cultures of *Agrobacterium* carrying pTRV_1_ and pTRV_2_ derivatives were grown overnight at 28 °C in Luria–Bertani (LB) medium supplemented with 50 mg/L kanamycin and 200 µM acetosyringone. The *Agrobacterium* were harvested using centrifugation (3000× *g*, 10 min, room temperature) and the pellets resuspended to an OD_600_ of 6 or 1 (for vacuum infiltration) in fresh inoculation buffer. The inoculation buffer contained 10 mM 2-[N-morpholino] ethane sulfonic acid (MES) (pH 5.5), 200 µM acetosyringone, 10 mM MgCl_2_, and sterile double-distilled water (DDW). *Agrobacterium* carrying pTRV_1_ [27,28,29] was then mixed with *Agrobacterium* carrying pTRV_2_ derivatives in a 1:1 ratio. The mixture was incubated for an additional 3 h at 28 °C with shaking at 200 rpm. Following this, 3-week-old plantlets were inoculated (at least 5 plants for each construct) by infiltration of the *Agrobacterium* mixture to the abaxial side of 3–4 leaves from each plant using a 1 mL needleless syringe; in addition, injections were made into the stems using a needle. As a negative control, plants were inoculated by pTRV_2_ (Figure 1d). 

For vacuum infiltration, 3-week-old plantlets were placed upside-down, with the leaves and stem submerged in the mixture of *Agrobacterium* inoculation buffer supplemented with 0.02% Tween 20 and placed in a vacuum chamber connected to a vacuum pump (Welch; Fürstenfeldbruck, Germany). Three cycles of vacuum were applied. Each cycle included the application of the vacuum for a period of 7–10 min until bubbles stopped appearing in the inoculation buffer. The vacuum was then slowly released by opening the valve. As negative controls, plants were mock inoculated (Figure 3n,q), or inoculated by pTRV_2_ (Figure 1d, Figure 2e, and Figure 3h,k). As a positive control for pTRV_2_-*DsRed* inoculation, *Nicotiana benthamiana* plants were inoculated in parallel to the cannabis plants (Figure 3b,c). 

### 3.4. RNA Extraction, cDNA Synthesis, and Quantitative Real-Time PCR (qRT)

RNA was extracted from cannabis leaves (2 weeks after infection) using a Bio-Tri Reagent kit (Bio-Lab Ltd., Jerusalem, Israel) according to the manufacturer’s instructions. First-strand cDNA was synthesized using a High-Capacity cDNA Reverse Transcription kit (Thermo Fisher Scientific, Waltham, MA, USA), 1 μg of total RNA following RNase-free DNase treatment (Promega, Madison, WI, USA), and a mixture of oligo (dT) and random hexamer primers. Primer sets 3 and 4 (Table 1) were used for TRV_2_ and TRV_2_-*DsRed* detection, respectively. PCR was performed using PCRBIO HS Taq (PCR Biosystems, London, United Kingdom) for 40 cycles (94 °C for 60 s and then cycling at 94 °C for 15 s, 60 °C for 15 s, and 72 °C for 60 s).

To evaluate *PDS* transcript levels, real-time qPCR was performed using 40 cycles (94 °C for 20 s and then cycling at 94 °C for 3 s and 60 °C for 30 s) in the presence of PowerUp SYBR Green Master Mix (Thermo Fisher Scientific, Waltham, MA, USA) on a StepOnePlus cycler (Thermo Fisher Scientific, Waltham, MA, USA). Relative expression levels were normalized to *UBQ5* as the reference gene [33] and calculated according to a standard curve generated for each gene using dilutions of cDNA samples. The real-time PCR primers used for *PDS* and *UBQ5* amplification were primer sets 5 and 6, respectively. Data analysis was performed using StepOne software version 2.2.2 (Thermo Fisher Scientific, Waltham, MA, USA).

### 3.5. Imaging

A stereoscopic fluorescent microscope Nikon SMZ25 (Nikon, Melville, NY, USA) equipped with a Leica DC300FX camera (Leica Microsystems, Buffalo Grove, IL, USA) was used for imaging.

## Figures and Tables

**Table 1:** The list of primers used in this study.

Primer Number	Product	Forward Primer	Reverse Primer
1	*PDS*_424_	5′-AATTCTCGAGCTTCAGCTCCCACCAGAGTC-3’	5’-ATTCTAGATCACCGTCATCATCTTTCCA-3’
2	*PDS*_486_	5’-AATTCTCGAGACTGGAAAGAGATTCCGTATTTCA-3’	5’-ATTCTAGAACAAAACCGCACCTTCCAT-3’
3	TRV_2_-*CP*	5′-ACGATTCTTGGGTGGAATCA-3′	5′-TCGTAACCGTTGTGTTTGGA-3′
4	TRV_2_-*DsRed*	5′-ACGATTCTTGGGTGGAATCA-3′	5′-CCCATGGTCTTCTTCTGCAT-3′
5	*PDS*	5′-ACTGTTCCTGATTGCGAACC-3′	5′-CTCGGCCAAAATTCTCTGAC-3′
6	* UBQ5 *	5′-AAGCTCGCTCTTCTCCAGTTC-3′	5′-CACACTTGCCGCAGTAATGTC-3′

## Data Availability

The data presented in this study are available in the article.
